# Efficacy of an Online Self-Help Treatment for Comorbid Alcohol Misuse and Emotional Problems in Young Adults: Protocol for a Randomized Controlled Trial

**DOI:** 10.2196/11298

**Published:** 2018-11-01

**Authors:** Jona R Frohlich, Karli K Rapinda, Michael P Schaub, Andreas Wenger, Christian Baumgartner, Edward A Johnson, Roisin M O'Connor, Norah Vincent, Matthijs Blankers, David D Ebert, Heather Hadjistavropoulos, Corey S Mackenzie, Matthew T Keough

**Affiliations:** 1 Department of Psychology University of Manitoba Winnipeg, MB Canada; 2 Swiss Research Institute for Public Health and Addiction University of Zurich Zurich Switzerland; 3 Department of Psychology Concordia University Montreal, QC Canada; 4 Clinical Health Psychology University of Manitoba Winnipeg, MB Canada; 5 Arkin Mental Health Care University of Amsterdam Amsterdam Netherlands; 6 Trimbos Institute Netherlands Institute of Mental Health and Addiction Utrecht Netherlands; 7 Academic Medical Center Department of Psychiatry University of Amsterdam Amsterdam Netherlands; 8 Department of eMental Health University of Erlangen-Nuremberg Erlangen Germany; 9 University of Regina Psychology Regina, SK Canada

**Keywords:** alcohol misuse, anxiety, cognitive behavioral therapy, depression, integrated treatment, mobile phone, motivational interviewing, online, self-help

## Abstract

**Background:**

Alcohol misuse and emotional problems (ie, depression and anxiety) are highly comorbid among Canadian young adults. However, there is a lack of integrated, accessible, and evidence-based treatment options for these young adults.

**Objective:**

The main goal of this study is to develop and test the efficacy of an integrated, online self-help program designed to target both alcohol misuse and emotional problems.

**Methods:**

A two-arm randomized controlled trial design will be used to compare the efficacy of the online integrated treatment to a psychoeducational control group. A target sample of 214 participants will be recruited and randomly assigned to either condition. The integrated treatment will last 8 weeks, and participants will work through 12 modules. Modules will incorporate content based on principles of cognitive behavioral therapy and motivational interviewing. Participants in the control group will receive links to psychoeducational resources and will have access to the full treatment after follow-up. The primary outcome will be the number of Canadian standard drinks consumed in the week leading up the assessment. Secondary outcomes of interest include symptoms of depression, anxiety, alcohol-related problems, quality of life, and use of other drugs. Assessments will be completed at 3 time-points: at baseline, at the end of treatment (ie, 8 weeks), and at follow-up (ie, 24 weeks). Upon completion, data will be analyzed using generalized linear mixed models.

**Results:**

Data collection began in June 2018 and will continue until January 2020. Final study results will be submitted for publication by July 2020.

**Conclusions:**

Currently, there are no integrated treatments designed to target alcohol misuse and the range of emotional problems experienced by young adults. This research stands to provide an effective, accessible (ie, Web-based), and feasible option to treat the many struggling young adults in this country.

**Trial Registration:**

ClinicalTrials.gov ID NCT03406039; https://clinicaltrials.gov/ct2/show/NCT03406039 (Archived by WebCite at http://www.webcitation.org/72fDefnrh)

**Registered Report Identifier:**

PRR1-10.2196/11298

## Introduction

### Background

Many young adult Canadians struggle with comorbid alcohol misuse and emotional problems (ie, depression and anxiety). According to national statistics, 3.1 million Canadians consume enough alcohol to place themselves at risk of harm or injury [1]. Existing reports show that 50%-60% of adults with an alcohol use disorder also meet clinical criteria for a depressive or anxiety disorder [2-5]. Prevalence rates of these mental health concerns vary based on the geographic location, with the highest rates of alcohol abuse and emotional problems being reported by people living in rural and Northern communities [1]. In addition, data show that the combinations of alcohol misuse and depression or anxiety are the most frequent mental health comorbidities in general populations [6,7]. We know that this comorbidity results in high disease burden—much more than each disorder separately [6,7]. At the individual level, comorbid alcohol misuse and depression or anxiety are linked to chronic poor health, increased suicidality, marital dysfunction, physical injury, and early mortality [2,8,9]. At a societal level, this comorbidity contributes to overburden on the health care system, high rates of absenteeism and unemployment, and increased legal problems [10]. There is currently a need for effective integrated treatments designed to treat alcohol misuse and co-occurring emotional problems.

This study will design and deliver an online self-help intervention for comorbid alcohol misuse and emotional problems in young adults (aged 18-35 years). This intervention will contain strategies drawn from cognitive behavioral therapy (CBT) and motivational interviewing (MI)—both evidence-based psychotherapies for alcohol misuse and depression or anxiety [11,12]. The main strength is that this intervention will be integrated—meaning that it will target symptoms of both alcohol misuse and depression or anxiety within the same treatment. In addition, the Web-based platform is advantageous relative to face-to-face approaches. We will be able to better reach individuals in rural and Northern communities—where we know that some of the highest rates of alcohol misuse and emotional issues exist. These individuals often have difficulty accessing in-person treatments because of community remoteness. Thus, they may be left to struggle with major mental health concerns and associated harms. Furthermore, young adults may feel less stigma associated with accessing support online. Thus, the proposed treatment has the potential to improve the health and well-being of young adult Canadians and their families.

### Current State of Knowledge

Research on integrated treatment is relatively new. This is surprising, given that it has been long understood that alcohol misuse and comorbid emotional problems are associated with a complex clinical presentation [5,8]. Specifically, the presence of an emotional disorder (relative to its absence) contributes to high rates of relapse, greater functional impairment, and poorer responses to treatment among individuals with substantial alcohol problems [5]. Traditional approaches to treating comorbid alcohol misuse and depression or anxiety include sequential or parallel intervention [13,14]. During a sequential approach, clinicians treat the disorder viewed as “primary” first, followed by the treatment of the comorbid condition. Often times, this means that symptoms of emotional problems are not addressed in treatment until an individual achieves some notable period of abstinence from drinking. Thus, in the sequential model, treatment is provided for one disorder at a time—with the more acute disorder (ie, alcohol misuse) taking first priority. The sequential model of intervention remains the most widely used approach to treating alcohol misuse-depression or anxiety comorbidities [14]. In contrast, the parallel model involves treating alcohol misuse and emotional problems separately by two distinct professionals or clinical teams [13,14]. A notable example of this approach would be a person seeing a family doctor for management of antidepressant medications while working with a psychologist to reduce drinking. Therefore, in the parallel model, an individual receives support for alcohol misuse and depression or anxiety simultaneously, but from distinct professionals.

Although still widely used, sequential and parallel approaches are limited as intervention models for comorbid disorders [14]. A sequential approach may be necessary for crisis situations, such as when a person needs hospitalization for alcohol-related seizures or acute suicidality. However, in the absence of an emergency warranting the immediate stabilization of one disorder over the other, sequential treatment may impede the treatment of both disorders [13]. Specifically, sequential treatment does not consider the interconnectedness of alcohol misuse and emotional problems. Moreover, in a parallel treatment model, there is often little communication between professionals independently treating each disorder [14]; this is problematic because professionals often have different case conceptualizations and treatment recommendations. Hence, it is very common for a person to receive conflicting advice and feedback in a parallel treatment approach [14]. Furthermore, it is up to patients to integrate distinct treatment approaches, which is likely difficult because of high rates of cognitive impairment among those with alcohol problems [14]. The limitations of a parallel approach may lead to adverse patient outcomes, such as frustration, continued mental health challenges, and, in the most extreme case, discontinuation of treatment of both disorders. Overall, attesting to the above limitations, the literature shows that sequential and parallel approaches result in poor treatment outcomes in those struggling with alcohol misuse and comorbid emotional problems [15-17].

Integrated psychological treatments are designed to target symptoms of comorbid disorders within the same intervention [14]. There is a growing interest in combining CBT and MI to treat alcohol misuse and comorbid emotional problems [6,18]. CBT is an evidence-based therapy where the goal is for patients to develop better coping skills for alcohol and mood or anxiety issues [11]. These skills become important when patients have to navigate challenging internal (eg, negative thoughts) and external (eg, stressors) triggers in their daily lives. Meta-analyses show that CBT leads to marked reductions in depression and anxiety symptoms, with effect sizes being moderate-to-large relative to other treatments [11,19]. In addition, relapse rates for depression after CBT tend to be half those of medication treatments [20]. Besides, CBT has been shown to be effective in reducing substance and alcohol use, though effect sizes tend to be smaller relative to the effects found for depression and anxiety [21]. Moreover, MI is an evidence-based psychological intervention, often described as a patient-centered, collaborative approach to help elicit and strengthen motivation for change [22]. The emphasis in MI is to help patients resolve ambivalence about change and, hence, move in a positive direction that is consistent with personal values. Several meta-analyses support the use of MI as a front-line intervention for alcohol misuse, with typical effect sizes being in the moderate-to-large range relative to no treatment [23]. Furthermore, MI has been used to treat depression and anxiety, though the evidence base is comparably smaller than for alcohol misuse [24,25].

Emerging work suggests that combined CBT and MI have synergistic, beneficial effects on both alcohol misuse and comorbid emotional problems within an integrated framework [6]. MI aims to improve patients’ motivation for changing problem behaviors. Motivational deficits are common in both alcohol misuse and emotional disorders, and the data show that low motivation for change predicts poor treatment engagement and poor outcomes [22]. By increasing motivation for change using MI, clients may be more willing to engage in the effortful activities of CBT (eg, homework and behavioral activation)—which are essential for building better coping skills. In addition, concurrent MI may help to clarify patients’ core values in CBT by developing a discrepancy between current and desired behavior. Thus, from a theoretical perspective, MI and CBT naturally complement each other in the treatment of comorbid alcohol and emotional problems. While this is a relatively new treatment approach, emerging data are promising. Much of the work has focused on testing the usefulness of CBT or MI for alcohol misuse and comorbid depression [6]. Comparatively, there has been much less focus on targeting co-occurring anxiety symptoms in this literature. A recent meta-analysis of 12 RCTs showed that combined integrated CBT or MI (relative to control conditions) reduces alcohol misuse and depressive symptoms [6]. Effect sizes were comparable for subclinical (relative to clinically elevated) symptoms, suggesting that integrated CBT or MI may be beneficial in earlier stages of risk as well. Furthermore, combined CBT or MI has the potential to alleviate co-occurring anxiety symptoms in those with alcohol misuse. However, the usefulness of integrated treatments for the alcohol misuse-anxiety comorbidity remains unexamined.

### Rationale and Objectives of the Proposed Study

Current integrated psychological treatments use a traditional face-to-face modality [6]. However, there are distinct advantages of online delivery of integrated treatment among Canadian young adults. First, online interventions would be able to reach young adults from rural and Northern communities. We know from most recent health statistics that the highest rates of alcohol misuse and emotional issues exist in these communities [1]. Second, young adults may be more willing to engage in an online self-help intervention (relative to in-person); this is because an online modality may be associated with reduced shame and stigma—which are known, persistent barriers to seeking treatment among substance abusers [26]. Third, online treatments offer the potential for early intervention [27,28]. Self-guided online interventions may be helpful during a period earlier in the risk pathway when young adults are experiencing moderate (but still subclinical) problems. This may serve to prevent an escalation of clinical disorders later in adulthood. Finally, online interventions could markedly reduce the burden on the mental health care system in Canada. More young people with alcohol misuse and co-occurring emotional problems would be helped for much less cost relative to inpatient treatments. Data show that cost-effective, online interventions reduce alcohol misuse [29], depression [30], and anxiety [31] separately, with effect sizes being comparable to those of in-person treatments.

The goal of the proposed study will be to adapt, implement, and test an integrated, online treatment for alcohol misuse and comorbid emotional problems in young adult Canadians. The intervention will be adapted from a new online integrated treatment developed by collaborators Schaub et al at the Swiss Research Institute for Public Health and Addiction [27] called *Take Care of You*; this intervention is in German and Dutch, and it is the first online integrated treatment for alcohol misuse and depression in young adults. *Take Care of Me* (ie, the current intervention) is an internet-based, self-help program designed to reduce symptoms of alcohol use and emotional problems (ie, depression and anxiety). The treatment combines elements of CBT and MI and is designed to target young adults at risk for developing more severe alcohol misuse, depression, and anxiety should they not receive care. This intervention will be translated into English, and new treatment modules will be added to target anxiety symptoms as well. This is an important and novel expansion of the German-Dutch intervention, given the high co-occurrence of alcohol misuse and anxiety symptoms in clinical and general populations [7,8]. In addition, depression and anxiety symptoms tend to co-occur at high rates in young adults [6]. Accordingly, the proposed study will be a randomized controlled trial (RCT) examining the efficacy of an online, self-help intervention for alcohol misuse and comorbid emotional problems relative to a psychoeducational control group. We have proposed the following hypotheses:

1. Participants in the integrated treatment condition will show larger reductions in weekly alcohol use (primary outcome) relative to participants in the psychoeducational control group over the 8-week *Take Care of Me* program.
2. Participants in the integrated treatment condition will show larger reductions on measures of alcohol misuse, depression, anxiety, as well as increases in a measure of the quality of life (secondary outcomes) over the 8-week *Take Care of Me* program.
3. Symptom improvements during integrated treatment will be maintained at 6-month follow-up.

## Methods

### Study Design

A two-arm RCT will be used. Eligible participants will be randomized to either the online integrated treatment condition or the psychoeducational control condition. Assessments will occur before randomization (T0; baseline), at 8 weeks (T1; treatment end), and at 24 weeks (T2; follow-up). The study will not be blinded. Participants will be made aware of which condition they have been assigned to as soon as they complete baseline measures and are deemed eligible. Blinding of researchers is not necessary as they will not be providing treatment to participants.

### Recruitment

Recruitment of study participants began in June 2018 and will continue until January 2020. Participants will be recruited through a number of means across the country (Textbox 1). Our main recruitment objective is to recruit across Canada using Web-based advertisements (eg, Google Ads, Kijiji, and Craigslist), contacting student services at major Canadian universities, and through social media (eg, Facebook). The goal is that utilizing Web-based means will ensure that a large sample of Canadian young adults receives the link to the study website. In addition, prominent addiction community services in the province of Manitoba have agreed to assist with recruitment, which will provide access to many individuals struggling with alcohol use problems. Study posters will also be put up at community organizations, hospitals, local university campuses, and popular drinking locations (eg, bars and restaurants). Recruitment rates will be monitored on an ongoing basis (eg, % completing screening relative to % meeting criteria to participate). With regard to our recruitment strategies, one potential drawback is that participants may come primarily from the same place (eg, the same university), creating a homogenous sample. However, our plan is to utilize many different avenues, with the goal of creating as heterogenous a sample as possible. Similarly, it is possible that those recruited through social media may share characteristics that interact with their experience in the program. Utilizing multiple recruitment strategies simultaneously, as well as examining potential moderators upon data analysis, will help mitigate this potential challenge.

### Inclusion and Exclusion Criteria

There has been a shift to include heterogeneous samples in RCTs to improve the generalizability of findings to other samples [32]. Therefore, inclusion or exclusion criteria will be kept to a minimum. Our main participant demographic will be young adult Canadians with, at least, moderate alcohol misuse and emotional symptoms. Young adults will be targeted as the highest national rates of drinking are observed among individuals in this age group [33]. Furthermore, we know young adults are at an increased risk of both alcohol-related problems and emotional challenges [6]. Inclusion criteria for participation will be as follows: (1) individuals aged 18-35 years; (2) scores >3 for women and >4 for men on a brief version of the Alcohol Use Disorders Identification Test (AUDIT-C) [33]; (3) self-reporting, at least, moderate depression or anxiety symptoms, or both, indicated by a score >16 on the Center for Epidemiological Studies Depression Scale (CES-D) [34], and a score of >10 on the Generalized Anxiety Disorder Scale (GAD-7) [35]; (4) fluency in English; and (5) having weekly internet access. Individuals will be ineligible to participate if they (1) self-report engaging in other psychological or pharmacological treatments for alcohol misuse or depression or anxiety; (2) report elevated suicidality, defined as scoring greater than “minimal risk” on the P4 Suicidality Screener [36], a widely used measure in RCTs, as well as in primary care settings worldwide; and (3) report current psychosis or mania.

### Informed Consent Procedure

**Figure 1:**
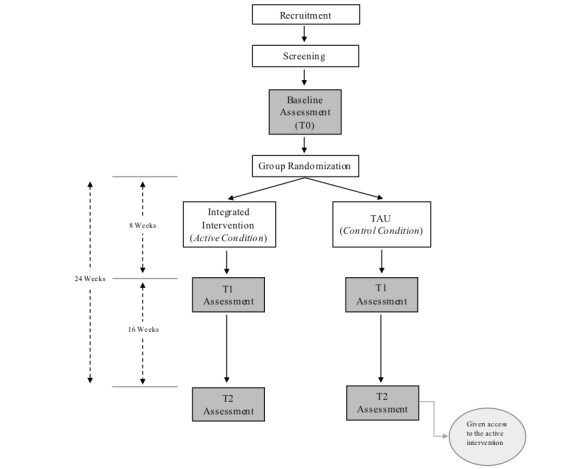
Randomization and trial flowchart. TAU: treatment as usual.

Interested participants will be invited to contact the primary investigators for a link to the study website (Figure 1). They will have seen this contact information from any of the recruitment materials that informed them about the study. The website link will include all pertinent information about the study; it will also direct them to the page where they will be able to provide informed consent on the treatment website. Prior to consenting, participants will be informed about the following: the inclusion or exclusion criteria of the study, the potential risks or benefits of completing the intervention, safety arrangements during and after the study, and that participation is voluntary. In addition, they will be told the circumstances under which they should contact their family doctor or another professional from an emergency list that will be accessible at all times via the menu item “Help Me” on the intervention website. They will first be provided with detailed instructions on how to create a user account for the self-help program and be encouraged to contact the researchers should they have any trouble. Informed consent will be demonstrated by checking several boxes, stating that they have read and understood the terms of the research. After participants have provided informed consent, they will be able to register on the study website. They will need to create a username as well as verify their account via email before they can access the website. All data will be encrypted and stored on a Canadian host server. No other personal information will be required to register. After informed consent has been obtained and their account has been verified, they will immediately be asked to complete the baseline assessment measures to determine the eligibility.

### Randomization and Trial Flow

Eligible participants will be randomly assigned to either the treatment or control condition using a 1:1 ratio (Figure 1); this will happen automatically on the study website.

Participants will immediately be provided with instructions on the study website once they have been assigned to either group. Participants assigned to the treatment condition will be told that they have been selected to participate in the program and will be provided with instructions on how to proceed. Individuals assigned to the control condition will be provided links to general psychoeducation websites about alcohol and mental illness but told that they would be given full access to the program in 6 months. Assessments will then take place at the end of treatment (ie, T1, 8 weeks) and at follow-up (ie, T2, 24 weeks or 16 weeks after the end of treatment). In addition, participants will be asked to provide brief feedback (eg, helpfulness and enjoyment) about their experience in the program at the follow-up assessment. Participants will be reminded of the assessments at each time-point via email. They will receive automatic reminders about the assessment every 2 days for 1 week until they have completed the assessment. Participants will be compensated with a Can $10 Amazon gift card for each assessment that they complete, for a total potential compensation of Can $30. Participants will be told about the compensation process during informed consent.

**Wording to be used in recruitment emails and advertisements.**

**Recruitment email**

Dear Participant,

You are receiving this e-mail because you may be eligible to participate in an online study being offered to young adults in Canada between the ages of 18 to 35 struggling with alcohol use and anxiety or depression?

The purpose of this study is to test the effectiveness of an 8-week, self-directed online treatment program designed to help you cope with symptoms of alcohol use and anxiety or depression. Over the course of the 8-weeks, you will learn strategies and techniques from 12 different modules to help you cope with these challenges, while also receiving online support from our researchers. Our hope is that by the end of the program, you will experience a reduction in your alcohol use as well as your symptoms of anxiety or depression. During the study, all information you provide will be kept confidential. You will also receive a small honorarium for your participation.

If this sounds like something you would be interested in, please visit the study website at www.takecareofme.ca to sign up. You may also contact either Jona Frohlich or Dr. Matthew Keough at support@takecareofme.ca. This research has been approved by the University of Manitoba Psychology/Sociology Research Ethics Board.

Thank you for your consideration!

*NOTE: You must be fluent in English, have weekly access to the internet, and not currently be in treatment elsewhere in order to participate. You must also be prepared to commit approximately 3 hours/week to the program.

**Advertisement**

RESEARCH STUDY

Are you between the ages of 18-35 and currently struggling with alcohol use and anxiety or depression?

If so, you may benefit from joining our treatment study about alcohol misuse and emotional problems in young adult Canadians.

You will be invited to participate in an 8-week, self-directed online treatment program designed to help you cope with symptoms of alcohol use and anxiety or depression. The program consists of 12 modules.

While participating in the online program may be challenging at times, it may also help reduce your alcohol use, as well as improve your emotional well-being. All of the information you provide during your participation in this study will remain confidential. You will also receive a small honorarium for your participation. *NOTE: You must not currently be in treatment elsewhere and be prepared to commit approximately 3 hours/week.

If you have any questions about this research, or for more information, please contact: Dr. Matthew Keough OR Jona Frohlich. Email: support@takecareofme.ca

This research has been approved by the University of Manitoba Psychology/Sociology Research Ethics Board. Concerns can be directed to the Human Ethics Coordinator @ 204-474-8113 or email: humanethics@umanitoba.ca

#### Integrated Treatment (Active Condition)

**Figure 2:**
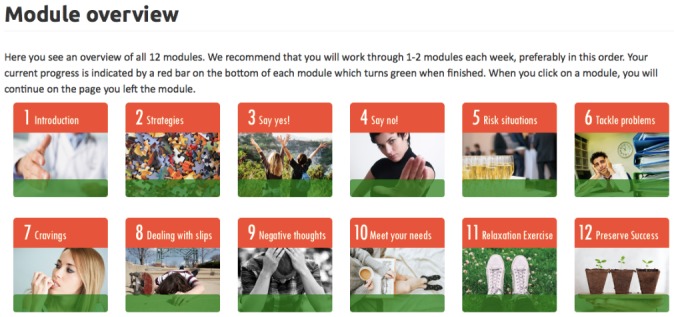
Main menu of intervention modules. Source: takecareofme.ca. Author: Swiss Research Institute for Public Health and Addiction.

The website content is a further development of the *Take Care of You* intervention [27] designed for use in English, with added content specific to symptoms of both depression *and* anxiety. The study website includes a dashboard with user information, a diary for participants to track their alcohol use and mood, 12 treatment modules, and a “Help Me” page of additional resources in various Canadian communities. Participants in the treatment condition will have access to 12 treatment modules (Figure 2) and will have 8 weeks to complete the modules. The average amount of text in each module is roughly the same. The content of all modules will be derived from CBT and MI principles (Table 1). For example, through module engagement, young adults will identify goals related to alcohol use and mood; learn strategies to cope with alcohol cravings, triggers, and social pressures; and learn how to prevent relapse. In addition, there will be content to target anxiety and depression, focusing on strategies designed to help reduce negative thinking and worry, increase behavioral activation, and increase self-care (eg, sleep hygiene). While participants can technically access all modules at once, they will be encouraged to work through the modules in sequential order. More specifically, they will be encouraged to complete 1-2 modules per week over the course of 8 weeks. However, they will be able to return to modules more than once or move ahead to modules that may be more relevant in a given moment if they so choose. For example, a participant may jump ahead to the relaxation module if he or she is having strong anxiety symptoms in a given week. Participants will be encouraged to complete the modules as many times as needed, and their progress will be visible on a digital progress bar. Modules will keep the place of participants within a module should they exit the program but want to continue where they left off upon their return. Furthermore, participants will also be asked to track their alcohol use and depression or anxiety symptoms each week. On the “dashboard” intervention page, participants will be able to see a graph depicting treatment progress. The website will automatically adapt for use on smartphones and tablets.

##### Intervention Support Person

The literature shows that adherence to treatment tends to be suboptimal in addiction intervention studies, including both in-person and online modalities [37]. In this study, comorbid emotional symptoms may compound potential issues with treatment adherence. For example, depression is associated with low motivation, and this, in turn, may contribute to difficulties with treatment engagement and homework completion. The support-accountability module [38] posits that these difficulties with adherence, particularly in online programs, may be mitigated by the support of an individual who is viewed as competent, trustworthy, and available. Therefore, an intervention support person (ie, a research assistant) will provide primarily automatic ongoing feedback about module progress, with reminders about completing sections of the intervention. This automatic feedback will also include motivational content that is personalized and signed off by the intervention support person. Textbox 2 shows the examples of this automated motivational content for the first few weeks. Emails of this nature will be sent for the duration of the active treatment condition. Moreover, a research assistant involved in this component of the project will provide support on demand—meaning that they will provide answers to questions when asked specifically by participants through email. Therefore, the use of a research assistant for this purpose will help to reduce the risk of attrition. Consistent with previous research, automatic and personal feedback should also improve adherence, as is commonly observed in self-guided behavior change interventions [38]. It is important to note that the purpose of the intervention support person (ie, research assistant) in this role is not to take the place of a therapist, but rather to answer any questions participants may have as they work their way through the modules, which will be largely administrative. Despite the anticipated benefits of incorporating support of this nature, it is overall not considered equivalent to receiving in-person therapy. However, we will obtain data on the number of emails exchanged for participants in each condition.

##### Dashboard

The purpose of this page is to provide a main menu where participants can access important information about the program and their progress in one place. On this page, they will be able to see the latest automated message from the research assistant, Deborah, the date on which they started the study, which assessments have been completed, their module progress, and their weekly mood and drinking diary. Furthermore, the dashboard will have an activity portal where participants can log an activity and corresponding mood if they wish.

##### Social Presence

Each module will begin with a brief introduction video to bring a small social presence to the treatment. Previous research suggests that adding this social factor will personalize the online program and may influence accountability [38], especially considering that it is self-guided. At the outset of treatment, participants will also be asked to select an animated personal companion whom they identify with in terms of sex, age, occupation, and family background. All companions will be fictional, yet relatable. The purpose of this companion is to provide advice and examples pertaining to the content throughout the intervention. The personal companion will appear in each module, at least, once.

##### Consumption and Mood Diary

Participants in the integrated treatment condition will be asked to track both their weekly alcohol use and mood as they progress through the program. These levels will be displayed on a graph that will be visible to participants at all times. Participants will be able to observe simultaneous changes in alcohol use and negative mood by consistently filling out their diary.

**Table 1:** Overview of the module content.

Module number and title	Module content
M1: Introduction	Introduction to intervention Motivational enhancement (ie, identifying reasons for change and pros and cons of drinking and not drinking) Self-monitoring alcohol use and mood
M2: Strategies for meeting your goals	Strategies to change drinking habits, including stimulus control (eg, ridding the home of cues) Resisting alcohol in specific situations (eg, situations involving negative emotions) Practicing refusal skills in high-risk situations Developing personal strategies to reduce or abstain from harmful alcohol use
M3: Say yes!	Learning about the relationship between mood and behavior Increasing behavioral activation (ie, scheduling rewarding or pleasurable activities during the week) Tips for dealing with motivational and implementation problems
M4: Learning to “say no” to alcohol	Learning about the various ways to say no to alcohol Ways to resist social pressures to drink Trying out a few role-plays to practice drinking refusal skills
M5: Identifying risky situations	Identifying personal high-risk situations for drinking Learning about “seemingly unimportant decisions” that could lead to heavy drinking
M6: Linking negative emotions and drinking to life problems	Relating issues with mood or alcohol to problems The difference between controllable and uncontrollable problems Formal problem solving (including problem identification, generating possible solutions, evaluating possible solutions, selecting a course of action, and evaluating the implementation of the action plan)
M7: Coping with craving	Psychoeducation about craving (eg, different forms of craving [mental and physical]) Introduce self-monitoring of craving New ways to effectively cope with cravings (eg, distraction, talking, experiencing the craving, and recalling the negative outcomes of drinking)
M8: Dealing with slips	Define a “slip” versus a full-blown relapse Introduce ways to cope with slip in mood and drinking
M9: Challenging automatic negative thoughts	Discuss think the link between thoughts, feelings, behaviors, and bodily sensations Review common thinking errors (or cognitive distortions) Introduce balanced thinking Encourage participants to make links between negative thoughts and drinking Encourage participants to complete at least one thought record for a problem situation Identity ways to “test” negative thoughts in real life
M10: Meeting your needs	Discussing “Sleep Hygiene” Ruminating and worrying less Increasing and improving social network
M11: Relaxation exercise	Key relaxation exercise to reduce anxiety (ie, progressive muscle relaxation) Deep breathing Scheduling relaxation times during the week
M12: Preserve your success	Identify “early warning signs” for slip or relapse Create personalized relapse prevention plan Discuss ways to cope with relapse Identify top five coping strategies

**Examples of automated motivational emails sent to the intervention group by the support person (ie, research assistant).**

**After 1 week**

Subject: First week is Over

Text:

Hi [Participant]!

You survived the first week! Congratulations! We hope you have been feeling well in the last few days.

Tomorrow starts your second week. Please log in today and fill out your diaries on . Fill in how much you drank in the past few days and how much you plan to drink in the upcoming week. Also, please enter your mood ratings for the previous week.

At this time, we would also like to encourage you to start another module if you haven’t yet done so. The more modules you do, the more likely you are to make progress!

Have a good week,

Deborah

**After 2 weeks**

Subject: Two weeks so far

Text:

Hello [Participant]

Two weeks have passed. We hope you are doing well and are starting to become familiar with the layout of this treatment program.

Tomorrow starts your third week. Please log in today and fill out your alcohol use and mood diaries on

Fill in how much you drank in the past few days and how much you plan to drink for in upcoming week. Also, Also, please enter your mood ratings for the previous week.

Have a good week,

Deborah

**After 3 weeks**

Subject: Week 3

Text:

Hello [Participant]!

You’ve completed three weeks of this course now! You’re doing great.

Maybe people in your life have noticed some changes in your behavior, (e.g. that you do not drink as much as you used to). If you encounter people trying to convince you to drink, we suggest you work through Module 4 (Say No).

And as always, don’t forget to update your drinking diary with your consumption for last week, as well as your intentions for next week on: [study website]. Also, please enter your mood ratings for the previous week.. Also, please enter your mood ratings for the previous week.

Have a good week,

Deborah

#### Psychoeducation (Control Condition)

Consistent with similar RCTs conducted in the field [6,27], the control group will receive links to websites that provide general psychoeducation about alcohol and mental illness. Therefore, the control group will be defined as having access to the available Web-based psychoeducational material. Participants assigned to the control group will be told that they cannot utilize the 12 modules right away, but that they can access the said resources in the meantime if they wish, and that they will be provided access to the full intervention at the end of follow-up (ie, 6-months after the first assessment). At both the end of treatment and follow-up, participants assigned to the control condition will be asked which resources they utilized and how many hours they spent, on average, looking at the said resources. This will help support the notion that both knowledge acquisition and time were controlled for (ie, differed between the 2 study arms).

### Measures

Table 2 provides a schedule of assessments. Demographic information will include age, biological sex, gender, ethnicity, history, and treatment for any physical or mental conditions, and family history of alcohol use. All self-report questionnaires will be administered through Web.

**Table 2:** Schedule of assessment measures.

Self-report measures	Baseline (T0)	8 weeks (T1)	24 weeks (T2)
Demographics (eg, age, sex, treatment history, psychiatric or medical history)	✓		
Suicidality (P4 Screener)	✓	✓	✓
Timeline Followback	✓	✓	✓
Center for Epidemiological Studies Depression Scale	✓	✓	✓
Generalized Anxiety Disorder Scale	✓	✓	✓
Alcohol Use Disorder Identification Test	✓	✓	✓
Quality of Life (World Health Organization Quality of Life brief version)	✓	✓	✓
Drug Use (National Institute on Drug Abuse Alcohol, Smoking, and Substance Involvement Screening Test)	✓	✓	✓
Motivation assessment	✓	✓	✓

#### Primary Outcome

Alcohol use will be assessed using the Timeline Followback (TLFB) procedure at all assessment points (T0-T2). The primary outcome measure will be the number of Canadian standard drinks consumed in the past 7 days prior to the assessment. Standard drinks are defined as a 12-oz can or a bottle of beer, a 5-oz glass of wine, or a 1.5-oz shot of hard liquor. The TLFB procedure has been shown to provide reliable and valid estimates of alcohol use and is widely used in basic [38] and treatment [27] studies.

#### Secondary Outcomes

##### Depression

Depression symptoms will be captured at all assessment points (T0-T2) using the CES-D, a 20-item self-report questionnaire. A sum score will be used. The CES-D is one of the most widely used, validated measures of depression severity [34].

##### Anxiety

Anxiety symptoms will be captured at all assessment points (T0-T2) using the GAD-7. The GAD-7 is a 7-item self-report instrument that measures the severity of anxiety symptoms. A sum score will be used. Studies in the extant literature support its reliability and validity [37].

##### Alcohol Misuse

The AUDIT is a 10-item self-report measure designed to assess alcohol misuse and will be included in this study as an additional secondary outcome. Overall, the measure provides an indication of both alcohol use and alcohol-related problems. The AUDIT is a widely used, reliable, and valid estimate of alcohol misuse [33] and will be administered at all assessment points (ie, T0, T1, and T2).

##### Combined Reduction of Alcohol Use and Comorbid Emotional Problems

We will also use a combined outcome to look at clinically significant reductions in alcohol misuse and depression or anxiety. Specifically, a short version of the AUDIT (first 3 items of the full AUDIT, referred to as AUDIT-C) will be used, where scores <3 for women and <4 for men would reflect that participants are no longer drinking hazardously. Similarly, falling below 16 on the CES-D or 10 on the GAD-7 would reflect that participants are no longer experiencing moderate emotional symptoms. A binary outcome will be created for participants scoring below the AUDIT-C cutoff and the cutoff for either depression or anxiety (coded as 1) versus those scoring above cutoffs (coded as 0). Furthermore, the AUDIT-C will be used to determine eligibility during the baseline assessment.

##### Quality of Life

An additional secondary outcome will be the quality of life. The World Health Organization Quality of Life Assessment (WHOQOL-BREF) is a 26-item self-report measure that assesses functionality in various life domains (eg, “How satisfied are you with your ability to perform your daily living activities?”). A sum score will be used to assess the overall quality of life at all assessments (T0-T2). The WHOQOL-BREF has become one of the most widely used quality of life measures in the literature [39].

##### Drug Use

Participants’ use of other drugs in addition to alcohol will also be included as a secondary outcome and assessed using the National Institute on Drug Abuse Alcohol, Smoking, and Substance Involvement Screening Test [40]. Examples of these additional substances include cannabis, cocaine, prescription medication, methamphetamine, and opioids. This information will be collected to examine potential transfer effects to other substances.

##### Motivation

Finally, given the large MI component, participants’ level of motivation will be assessed at all assessment points. The motivation for change will be assessed along 3 dimensions—importance, confidence, and readiness. Participants will be asked how important it is, how confident they are, and how ready they are to change their alcohol use and negative emotions. Each item will be assessed using a single item on a scale ranging from 0 *(Not Important or Confident or Ready)* to 10 *(Very Important or Confident or Ready)*.

### Sample Size Calculation

A recent meta-analysis showed that combined CBT and MI resulted in statistically significant reductions in alcohol use and depressive symptoms, with effect sizes being in the small range for both clinical and subclinical groups (*g*=0.17-0.27). Data show relatively comparable effect sizes for online and in-person interventions for alcohol misuse and emotional symptoms [29,30]. Therefore, a small effect size (*g*=0.25) is expected for group differences in alcohol use and emotional symptoms at the end of treatment. Using G*Power, the sample size required to detect a small effect with 80% power, alpha=.05, and a correlation of .50 between repeated measures was N=164. Based on the previous literature using online interventions to target alcohol use and depression [28], approximately 30% of participants are expected to be lost at follow-up. Therefore, to consider attrition, the sample size will be increased to N=214; this will allow for a buffer, should recruitment and attrition pose more of a challenge than anticipated.

### Data Analysis Plan

Before hypothesis testing, we will run preliminary analyses (eg, baseline differences and missing data analyses). In addition, we will examine overall group differences in clinically meaningful variables (ie, % below moderate cutoffs on the AUDIT, CES-D, and GAD-7 after treatment and at follow-up). Next, generalized linear mixed models will be used under the framework of intent-to-treat to evaluate the main hypotheses that integrated treatment will result in the largest reductions in alcohol use, depression, and anxiety. The use of generalized linear mixed models is a multilevel modeling technique that is preferable to repeated-measures analysis of variance for pre- and postanalyses, as it will allow us to include all randomized participants in analyses [41]. This results in less bias due to missing follow-up data [41]. However, owing to potential attrition problems, we also plan to conduct a sensitivity analysis to ensure that data are missing at random. Furthermore, primary and secondary outcomes will be tested sequentially in a mixed within- (repeated assessments) and between-subjects (treatment condition) design.

### Safety

We are aware of the increased risks associated with recruiting young adults with moderate (and distressing) alcohol misuse and emotional symptoms. We would expect higher base rates of suicidality and self-harming behaviors in these individuals relative to those without these mental health concerns. Accordingly, we will have safeguards to minimize the risk of harm. Young adults who report suicidal ideation and plans during screening will be recommended to visit their local medical professional or hospital for support. In addition, they will be given access to the integrated intervention, but their data will neither be analyzed nor included in the RCT. We will also be monitoring changes in suicidality at each assessment and will direct participants to emergency services if needed. Moreover, participants will have full-time access to a list of mental health services, including community resources, hospitals, and helplines. Supports will also be accessible to participants at all times via the menu item “Help Me” on the intervention website. Finally, participants in the control condition will be given access to the active treatment after the final assessment (6 months). They will receive all elements of the integrated intervention. To maintain transparency, updates will be made on ClinicalTrials.gov at each stage of the research process. Finally, the project has been funded by Research Manitoba, meaning that it has undergone a substantial peer-review process also considering safety aspects right from the beginning (Multimedia Appendices 1 and 2).

## Results

The intervention will be designed to adhere to the ethical principles of the Declaration of Helsinki and the Consolidated Standards of Reporting Trials guidelines for internet-based interventions [42]. The study has been granted procedural ethics from the Psychology/Sociology Research Ethics Board at the University of Manitoba, P2017:128 HS21125. All study procedures will be conducted in accordance with this ethics board at the granting institution, who will receive updates of the study status and will immediately be notified when the study is complete. Finally, the intervention is currently registered on ClinicalTrials.gov for traceability (ID: NCT03406039). We will update the status of the intervention at each stage of the study process in accordance with website guidelines.

Primary trial findings will be published in open-access journals so that both the public and policy makers in Canada can learn about and use the intervention. Anonymized study data will be available on request, and interested participants will be provided with a summary of the results once the study is complete. Finally, dissemination will also include providing talks at various community locations, major hospitals and universities, and at public institutions (eg, libraries) to ensure that results are as far-reaching as possible.

## Discussion

### Principal Findings

High rates of substance use and emotional issues in Canada demonstrate a clear need for additional and more accessible mental health services in the country. In addition, many provinces have considerable rural spread, meaning that several communities are dispersed throughout the province with little access to major city centers; this poses a substantial challenge for providing equal access to mental health care services. Furthermore, young people living in remote communities are at a marked disadvantage, as they seem to be struggling most with substance use and related problems but have limited access to treatment facilities. Thus, the proposed study has the potential to substantially improve the health and well-being of young adults living across the country. Given that we will be targeting young adults (ie, those who are early in the risk pathway), our intervention may help individuals change their mental health trajectories. That is, an early evidence-based intervention may provide “at-risk” young adults effective coping strategies and, therefore, prevent the escalation of problems in the future, thus, resulting in fewer adults with alcohol use and emotional disorders. Furthermore, the intervention stands to provide a cost-effective method (relative to traditional in-person treatments) of improving mental health care delivery to Canadians. Online interventions have the potential to save the government millions of dollars via the reduced burden on the health care system.

### Limitations and Mitigation Plan

Intervention studies show substantial variability in attrition rates, depending on the treatment orientation and clinical severity [43,44]. Generally, dropout rates are high in populations with alcohol and substance use disorders—ranging from 21% to 80% [45]. We are aware of this possible influence on attrition. However, regular contact with the intervention support person will likely provide personalized support that is not typical of online interventions; this may reduce dropout. In addition, we will offer participants the possibility to receive a summary report at the end of the study of overall findings. This report will contain information about the effects of the treatment, significant or not, based on aggregate-level data. The combined use of the strategies above will help reduce attrition. An additional limitation of this study is the use of self-report measures in a Web-based format, of which not all have been tested online. However, assessments of this nature remain widely used in clinical research and increasing reliability and validity evidence for these Web-based tools is accumulating in the clinical literature.

### Conclusions

The proposed intervention is a potentially effective, cost-friendly means to enhance mental health care delivery to young adults in Canada. Currently, no such treatments exist for young adults in the country who struggle with addiction and comorbid emotional problems. Research wise, the proposed intervention is a novel expansion of the Swiss study. One major new feature will be the inclusion of content that specifically targets anxiety symptoms—which are currently missing from existing online integrated treatments. It is essential to provide young adults with the skills to cope with these emotional problems because both are known triggers for alcohol misuse and both often occur within the same individual. Therefore, by including new skills to target anxiety, we will enhance the therapeutic benefits of our intervention. Overall, the proposed intervention stands to have a substantial positive impact on the lives of young Canadians and on government expenditures related to mental health care.

## Appendix

Multimedia Appendix 1 Grant review 1.

Multimedia Appendix 2 Grant review 2.

## Abbreviations

AUDIT-C: Alcohol Use Disorders Identification Test brief version
CBT: cognitive behavioral therapy
CES-D: Center for Epidemiological Studies Depression Scale
GAD-7: Generalized Anxiety Disorder Scale-7th edition
MI: motivational interviewing
RCT: randomized controlled trial
TLFB: Timeline Followback
WHOQOL-BREF: The World Health Organization Quality of Life Assessment brief version
