# Supplementary material for: Efficacy of an Online Self-Help Treatment for Comorbid Alcohol Misuse and Emotional Problems in Young Adults: Protocol for a Randomized Controlled Trial
Source: JMIR Res Protoc. 2018 Nov 1;7(11):e11298. doi: 10.2196/11298 (PMC6238101; doi:10.2196/11298)
Supplement: Multimedia Appendix 1 [file resprot_v7i11e11298_app1.pdf]

NEW INVESTIGATOR OPERATING APPLICATION

REVIEWER REPORT FORM

APPLICANT INFORMATION

**Project Number:**	2882
**Title of Project:**	Testing the Efficacy of an Online Self-Help Treatment for Comorbid Alcohol Misuse and Emotional Problems in Young Adult Manitobans: A Randomized Controlled Trial (RCT)
**Principal Applicant:**	Keough, Matthew

REVIEWER COMMENTS

Research Environment (Investigator)

appropriate, with funding support in early career. Collaborators strong mentoring resources.

Investigator(s) Experience

Strong clinical supervision record. Emerging supervision record that is more than adequate at this stage, with clear plan for developing increased student research supervision through grant support. RCT and intervention experience. Strong productivity for early career, relevant and respectable publication and funding record.

Project Overview

Strong project with clear rationale, clearly linked to program of research, and with strong metalworking collaborators. Project aims are appropriate for funding competition, and hold potential to improve health in MB.

Research Project/Program merit and Feasibility

Important research question, clear questions and methods. Clear indication of awareness of sensitive ethical issues and unique attrition potential, with clear back up plans. Novel aspects highlighted and clearly defended.

Demonstrated Use

Clear plan to extend and parlay into Tri-council funding, building on this project.

Gender and/or Sex-based analysis of research

Yes, considerations of sex and gender, clearly articulated and rationalized. unique approach to explore gender identify as it relates to substance use and mental health, which is novel and critical for research.

OVERALL COMMENTS

A strong proposal, clearly linked to research program, past experience,and demonstrated competence. Reasonable budget for a feasible project, with strong support for students. Strong publication and granting record for career stage, and strong collaborators as mentors, increasing feasibility and adding integrity to methodological expertise. Very important project, especially in light of current substance crisis issues, despite not focusing specifically on this particular issues. As such, this is a timely and important project with the potential not only to improve our knowledge base, but also to benefit participants.

Additional info would be helpful for budget items. In particular, the proposal suggests payment for a collaborator and I'm unsure if this is appropriate. It seems unusal, but i couldn't find much detail in the program guide to say this was a problem.
