# Supplementary material for: Efficacy of an Online Self-Help Treatment for Comorbid Alcohol Misuse and Emotional Problems in Young Adults: Protocol for a Randomized Controlled Trial
Source: JMIR Res Protoc. 2018 Nov 1;7(11):e11298. doi: 10.2196/11298 (PMC6238101; doi:10.2196/11298)
Supplement: Multimedia Appendix 2 [file resprot_v7i11e11298_app2.pdf]

Research Manitoba

NEW INVESTIGATOR OPERATING APPLICATION

REVIEWER REPORT FORM

APPLICANT INFORMATION

**Project Number:**	2882
**Title of Project:**	Testing the Efficacy of an Online Self-Help Treatment for Comorbid Alcohol Misuse and Emotional Problems in Young Adult Manitobans: A Randomized Controlled Trial (RCT)
**Principal Applicant:**	Keough, Matthew

REVIEWER COMMENTS

Research Environment (Investigator)

The Applicant

Dr. Matthew Keough is a recently recruited Assistant Professor in the Department of Psychology at the University of Manitoba. He began a tenure track position at UM in January 2017. His research interests have spanned fields of addictions and mental health, among youth and young adults. His methodological approach has been to explore the combined effects of coexisting / co-occurring problems, rather than to explore issues in a sequential fashion. His doctoral and post-doctoral experience in this domain has provided the experience to undertake the proposed research. He is currently working with a local team on co-existing alcohol abuse and problem gambling. The current project, funded by the Manitoba Gambling Research Program, is underway, and has similarities in design and protocol to the proposed research. The practical experience gained with the ongoing project will be a helpful guide. Further, successful completion of both projects should support a competitive application to national funding agencies.

Investigator(s) Experience

Collaborators are:
Dr Roisin O'Connor, Concordia University
Dr Edward Johnson, Department of Psychology at UM
Dr Norah Vincent, Clinical health Psychology at UM
Dr Michael Schaub, University of Zurich

All collaborators have supplied convincing, and positive, letters of support for Dr. Keough and for this proposal. To quote Dr Schaub, "Due to his past research in alcohol misuse and co-occurring emotional problems … Prof. Keough is the perfect candidate to lead the proposed intervention study."

Project Overview

This proposal is very well written.

A strength of this proposal is the clarity in description of the design and protocol for this RCT to examine the utility of a series of online modules aimed to help treat/reduce co-existing alcohol misuse and emotional issues of depression and anxiety among Manitoba young adults at moderate risk

Research Project/Program merit and Feasibility

The proposal builds on existing strength gained by this new investigator from his graduate and post-graduate education, as well as from the experience he is currently getting as a collaborator in a project with similar inquiry underway in Manitoba related to coexisting problem drinking and problem gambling.

The design of this project follows the protocol of an RCT, where the key intervention is an online program of 12 modules for the treatment of alcohol misuse and co-existing emotional issues. A feature of the plan, is that the targeted population is young "at risk" adults, who have access to web-based learning, but are physically living in remote and rural northern Manitoba locations. The applicant poses anticipated challenges, and provides solutions to them, suggesting this design is way to reach participants, where the feasibility of face-to-face counselling is otherwise limited. Participants will be able to engage with online e-coaches as they progress through the modules.

Participants will complete a battery of assessments / questionnaires at baseline, 8-weeks and 6 months. The usual care (Treatment as Usual) group will be offered the intervention at the end of the study. The detailed description of the protocol and data to be collected are clear. The statistical analysis is appropriate and well described.

Demonstrated Use

A convincing thread was described linking the applicant's previous training, including publications and presentations in this field, to the current proposal, and to strong potential for future research with secured national funding.

Gender and/or Sex-based analysis of research

The applicant has thoughtfully addressed “gender and sex based analysis” considerations.

OVERALL COMMENTS

A comment about the Budget for this proposal would be that close to half the requested funds will be going to Switzerland, to cover expense for the IT component, its translation and development, and to Dr. Schaub, the collaborator, and expert in online intervention studies. Pilot data from a similar study underway in Switzerland supports small but suggests positive effects for their online 3-arm intervention aimed at alcohol misuse and depression.

Overall, this is a very good proposal. It is clearly written, and has a high likelihood of successful completion, leading to gains in health for at risk Manitoba young adults in remote and northern locations.
